# Supplementary material for: Unusual dominant genotype NIA1 of Enterocytozoon bieneusi in children in Southern Xinjiang, China
Source: PLoS Negl Trop Dis. 2020 Jun 22;14(6):e0008293. doi: 10.1371/journal.pntd.0008293 (PMC7332067; doi:10.1371/journal.pntd.0008293)
Supplement: Attachment — Submitted filename: Review comments.docx [file pntd.0008293.s002.docx]

The results are some of novel and interesting, and the manuscript was well-organized. However, there are some issues that need to be resolved before consideration for publication.

1. This study conducted a cross-sectional survey of kindergarten children in some counties in southern Xinjiang, and found that the microsporidian genotypes which identified in HIV-positive or AIDS patients are the main infection genotypes, and other genotypes are also found. However, due to the lack of methodology and analysis of other children's infection influencing factors, it seems that the article content is less, needs to be supplemented and enriched

2. Table 2 describes the difference in gender infection, not the analysis of age differences in the text. In fact, the statistical difference analysis of these two indicators is not done.

3. It is recommended to add a map of the sample kindergarten.

曹老师，NTD审稿意见如下：1. 本研究对南疆部分县的幼儿园儿童做了横断面的调查，发现了平时仅在HIV阳性或AIDS患者中鉴定的微孢子虫基因型为主要的感染基因型，还发现别的基因型，但是由于方法学和儿童其他感染影响因素分析的缺乏，显得文章有些单薄，投稿NTD可能有点不够。2.Table2描述的是性别的感染差异，而不是文中所致的年龄差异分析，其实文中并没有做这两个指标的统计学差异分析；3.Table 1没有按统计学的三线表格制作方式；4. 建议补充一张采样幼儿园的地图。

总的评价：文章虽有一点新发现，但整体新意不足。

如果您给予大修的意见的话，那么就要求补充危险因素的分析，为什么这些地方这种基因型会那么高？

曹老师，NTD审稿意见如下：

1. 本研究对南疆部分县的幼儿园儿童做了横断面的调查，发现了平时仅在HIV阳性或AIDS患者中鉴定的微孢子虫基因型为主要的感染基因型，还发现别的基因型，

This study conducted a cross-sectional survey of kindergarten children in some counties in southern Xinjiang, and found that the microsporidian genotypes which identified in HIV-positive or AIDS patients are the main infection genotypes, and other genotypes are also found.

但是由于方法学和儿童其他感染影响因素分析的缺乏，显得文章有些单薄，投稿NTD可能有点不够。

However, due to the lack of methodology and analysis of other children's infection influencing factors, it seems that the article content is less, needs to be supplemented and enriched

2.Table2描述的是性别的感染差异，而不是文中所致的年龄差异分析，其实文中并没有做这两个指标的统计学差异分析；

Table 2 describes the difference in gender infection, not the analysis of age differences in the text. In fact, the statistical difference analysis of these two indicators is not done.

3.Table 1没有按统计学的三线表格制作方式；

Table 1 does not follow the statistical three-line form

4. 建议补充一张采样幼儿园的地图。

It is recommended to add a map of the sample kindergarten.

总的评价：文章虽有一点新发现，但整体新意不足。

Overall evaluation: Although the article has some new findings, the overall innovation is insufficient.

如果您给予大修的意见的话，那么就要求补充危险因素的分析，为什么这些地方这种基因型会那么高？
