# Supplementary material for: Unusual dominant genotype NIA1 of Enterocytozoon bieneusi in children in Southern Xinjiang, China
Source: PLoS Negl Trop Dis. 2020 Jun 22;14(6):e0008293. doi: 10.1371/journal.pntd.0008293 (PMC7332067; doi:10.1371/journal.pntd.0008293)
Supplement: Attachment — Submitted filename: response to PNTD-S-19-02284.docx [file pntd.0008293.s003.docx]

Response to PNTD-S-19-02284

Dear editor,

Thank you for the review of our manuscript “Unusual dominant genotype NIA1 of Enterocytozoon bieneusi in children in Southern Xinjiang, China”. We appreciate the constructive comments from you and the reviewers and we are pleased to enclose the revised manuscript and point-by-point responses to the comments of the reviewers. All the line numbers and page numbers refer to the tracked copy.

Sincerely yours

Longxian Zhang, Ph.D, Professor

Parasitology Laboratory

College of Animal Science and Veterinary Medicine

Henan Agricultural University

Zhengzhou 450002

China

REPONSE TO REVIEWERS’ COMMENTS

All the line numbers and page numbers refer to the tracked copy.

**Methods**

- Reviewer #1:

Keywords:

**Question 1:**

- Add: novel genotypes.

**Response:** Thank you very much. We have added “novel genotypes” to Keywords.

Abstract:

**Question 2:**

- Lines 16-17: “*Enterocytozoon bieneusi* causes approximately 90% of reported cases of human microsporidiosis”. Add: “mainly pathologies or intestinal disorders”.

**Response:** Thank you very much. We have revised this. Seen in Line 16.

Author summary:

**Question 3:**

- Lines 40-41: “Additionally, we demonstrated the zoonotic potential of E. bieneusi genotype D..”. Change: I prefer to use “we confirmed” or “we contrasted”.

**Response:** Thank you very much. We have changed “demonstrated” to “confirmed”. Seen in Line 45.

**Question 4:** - Comment: The authors must mention the groups 1 and 2 for grouping the nine identified genotypes and highlight the three novel genotypes.

**Response:** Thank you very much. We have revised this. Seen in Line 39 to 42.

Introduction:

**Question 5:** - Line 48: “may cause persistent diarrhea, vomiting and a wasting síndrome”. Add: “dissemination and presence in other organs”.

**Response:** Thank you very much. We have revised this. Seen in Line 53.

**Question 6:** - Lines 57-58. Comment: The zoonotic role of group 2 and mainly of group 1 should be highlighted. Finally, cite risk factors, sources of infection and animal hosts (reservoirs).

**Response:** Thank you very much. We have highlighted the zoonotic role of group 2 and mainly of group 1, Seen in Line 61 to 64. Risk factors was indicated in Line 54 to 56, and sources of infection and animal hosts was demonstrated in Discussion.

**Question 7:** - Line 64: “in China have been reported by several studies”. Add: references [6-14]?.

**Response:** Thank you very much. We have added the references. Seen in Line 71.

Materials and Methods:

**Question 8:** - Ethics statements: Lines 82-83. “Participants infected with parasites received 83 appropriate treatment according to local policies and national guidelines”. Comment: It is unknown if patients have an active infection or are spores passing through the intestine without causing a real infection in the patient. I prefer to remove “Participants infected” and put “Participants with possible presence of parasites” or “Positive participants in the study with parasites”.

**Response:** Thank you very much. We have removed “Participants infected” and put “Participants with possible presence of parasites”. Seen in Line 90 and 91.

**Question 9:** - Sample collection: Lines 86-87. In my opinion it would be very important to indicate if the counties studied are in urban or rural areas. This information would help a greater knowledge of the circulation of different genotypes according to the type of area and the presence or absence of animal hosts as a source of infection.

**Response:** Thank you very much. All these counties are in rural areas, and daily consumption of meat is mainly beef and lamb in these counties. Animal husbandry is the main economic industry.

**Question 10:** - Sample collection: Line 91. “No diarrhea was observed during sampling”. Comment: It should be indicated if children have any other signs or symptoms, for example, weight loss, malnutrition, fever, etc. or even some other associated pathology (if this would be possible).

**Response:** Thank you very much. At the design stage of this study the inclusion of these symptoms was considered. As an intestinal pathogen, the main symptom it causes is diarrhea. Therefore, the fecal collector was only marked with the date and the participant’s name, age, and sex. Other signs or symptoms will be included in our next further study.

- DNA extraction and PCR amplification. The authors should provide some additional nested PCR information. For example, the pairs of primers used, temperature cycles, independently of the bibliographic references.

**Response:** Thank you very much. We have added detailed nested PCR information, Seen in Line 115 to 125.

- Reviewer #2:

**Question 1:** 1.the sample collection is from 11 counties of Xinjiang ,China, suggest to use a map to fix location.

**Response:** Thank you very much. We have added a map indicating the sampling sites. Seen Figure 1.

**Question 2:** 2."the studies were conducted in diverse geographical locations (Jilin, Heilongjiang, Guangxi, Henan, Shanghai, Hubei, and Chongqing)", need to supply more references, such as PLoS Negl Trop Dis 13(5): e0007356. https://doi.org/10.1371/journal.pntd.0007356.

**Response:** Thank you very much. We have added this reference. Seen in Line 71, 73, 75 and 337 to 341.

- Reviewer #3: (No Response)

--------------------

Results

- Reviewer #1:

**Question 1:** Phylogenetic analysis: Lines 163-164. “(CHN6, CXJH2, and CXJH3) clustered together with the zoonotic genotypes I, J, and BEB6 (Figure 1)”. Add: group 2.

**Response:** Thank you very much. We have added this, Seen in Line 189.

**Question 2:** Table 1: Indicate the urban or rural character of the counties studied.

**Response:** Thank you very much. All the 11 counties involved in this study are rural counties, and we have added a map indicating the locations of these counties. Seen in Figure 1.

- Reviewer #2:

**Question 1:** 1.the line 141, I think that it make a mistake, "age groupe"? If so, please add it.

**Response:** Thank you very much. We have revised this, Seen in Line 167.

- Reviewer #3: (No Response)

--------------------

Conclusions

- Reviewer #1:

**Question 1:** The authors should modify the conclusions based on the new information provided in the Discussion.

For example:

- Line 201. Remember: In my opinion it would be very important to indicate if the counties studied are in urban or rural areas. This information would help a greater knowledge of the circulation of different genotypes according to the type of area and the presence or absence of animal hosts as a source of infection.

- The authors have recently published the following article that should be discussed in this section and included in the bibliography. “Li D-F, Zhang Y, Jiang Y-X, Xing J-M, Tao D-Y, Zhao AY, Cui Z-H, Jing B, Qi M and Zhang L-X (2019). Genotyping and Zoonotic Potential of Enterocytozoon bieneusi in Pigs in Xinjiang, China. Front. Microbiol. 10:2401. doi: 10.3389/fmicb.2019.02401”.

It is very important because the second genotype (D) and the fourth (EbpA) identified in children are those detected in pigs in the same geographical area of Xinjiang (China).

Therefore, it could reinforce the role or zoonotic character of some of the genotypes found in children and the importance of the geographical area (urban or rural).

**Response:** Thank you very much. We have revised this, Seen in Line 243 to 247, and 259.

- Reviewer #2:

**Question 1:** this study conclusions are proper. the data of analysis is correct. the discuss can help to understanding the topics.

**Response:** Thank you very much.

- Reviewer #3: (No Response)

--------------------

Editorial and Data Presentation Modifications?

- Reviewer #1: (No Response)
- Reviewer #2:

**Question 1:** the objectives of study are very clear, and the sdudy design is appropriate toaddress the objectives.

**Response:** Thank you very much.

- Reviewer #3: (No Response)

--------------------

**Summary and General Comments**

- Reviewer #1:

Discussion:

**Question 1:** - Line 184: “as in a study which found that 11 of 93 fecal specimens collected from children with”. Add: percentage (%).

**Response:** Thank you very much. We have revised this, Seen in Line 210.

**Question 2:** - Line 199: “dominant genotype in children in Southern Xinjiang in this study”. Add: percentage (%).

**Response:** Thank you very much. We have revised this, Seen in Line 225.

**Question 3:** - Line 201. Remember: In my opinion it would be very important to indicate if the counties studied are in urban or rural areas. This information would help a greater knowledge of the circulation of different genotypes according to the type of area and the presence or absence of animal hosts as a source of infection.

- Line 202: “Genotype D was the second most prevalent genotype detected in this study”. Add: percentage (%).

- The authors have recently published the following article that should be discussed in this section and included in the bibliography. “Li D-F, Zhang Y, Jiang Y-X, Xing J-M, Tao D-Y, Zhao AY, Cui Z-H, Jing B, Qi M and Zhang L-X (2019). Genotyping and Zoonotic Potential of Enterocytozoon bieneusi in Pigs in Xinjiang, China. Front. Microbiol. 10:2401. doi: 10.3389/fmicb.2019.02401”.

It is very important because the second genotype (D) and the fourth (EbpA) identified in children are those detected in pigs in the same geographical area of Xinjiang (China).

Therefore, it could reinforce the role or zoonotic character of some of the genotypes found in children and the importance of the geographical area (urban or rural).

**Response:** Thank you very much. We have revised this, Seen in Line 243 to 247, and 259.

References:

**Question 1:** - Add: Li D-F, Zhang Y, Jiang Y-X, Xing J-M, Tao D-Y, Zhao A-Y, Cui Z-H, Jing B, Qi M and Zhang LX (2019). Genotyping and Zoonotic Potential of Enterocytozoon bieneusi in Pigs in Xinjiang, China. Front. Microbiol. 10:2401. doi: 10.3389/fmicb.2019.02401.

**Response:** Thank you very much. We have added this reference, Seen in Line 400 to 403.

- Reviewer #2:

**Question 1:** This study reports the zoonotic potential of E. bieneusi genotype D . this study is also the first report of human infection by E. bieneusi genotypes KB-1 and CHN6 in China. This is an important significance .

**Response:** Thank you very much.

- Reviewer #3: (No Response)
