# Supplementary material for: Unusual dominant genotype NIA1 of Enterocytozoon bieneusi in children in Southern Xinjiang, China
Source: PLoS Negl Trop Dis. 2020 Jun 22;14(6):e0008293. doi: 10.1371/journal.pntd.0008293 (PMC7332067; doi:10.1371/journal.pntd.0008293)
Supplement: Attachment — Submitted filename: Comments.docx [file pntd.0008293.s004.docx]

The author has already addressed the relevant questions and the authors have made the changes. I have just a few additional comments.

1. “unusual dominant genotype NIA1” is missing in the abstract, please add it.

2. Line 59, ‘of which 96 have been…’ should be revised to ‘of which more than 96 have been…’

3. Line 213, healthcare level also should be included here.

4. Line 251, ‘…isolates identified in ruminants.’. Revised word ‘isolates’ to ‘genotypes’.
