# Supplementary material for: Unusual dominant genotype NIA1 of Enterocytozoon bieneusi in children in Southern Xinjiang, China
Source: PLoS Negl Trop Dis. 2020 Jun 22;14(6):e0008293. doi: 10.1371/journal.pntd.0008293 (PMC7332067; doi:10.1371/journal.pntd.0008293)
Supplement: Attachment — Submitted filename: response to PNTD-S-19-02284-R2.docx [file pntd.0008293.s005.docx]

Response to PNTD-S-19-02284

Dear editor,

Thank you for the review of our manuscript “Unusual dominant genotype NIA1 of Enterocytozoon bieneusi in children in Southern Xinjiang, China”. We appreciate the constructive comments from you and the reviewers and we are pleased to enclose the revised manuscript and point-by-point responses to the comments of the reviewers. All the line numbers and page numbers refer to the tracked copy.

Sincerely yours

Longxian Zhang, Ph.D, Professor

Parasitology Laboratory

College of Animal Science and Veterinary Medicine

Henan Agricultural University

Zhengzhou 450002

China

REPONSE TO REVIEWERS’ COMMENTS

All the line numbers and page numbers refer to the tracked copy.

**Editorial and Data Presentation Modifications**

**Question 1:** - Lines 16-17: “Enterocytozoon bieneusi causes approximately 90% of reported cases of human microsporidiosis”. Add: “mainly pathologies or intestinal disorders”. Response: Thank you very much. We have revised this. Seen in Line 16.

The authors must put all words “mainly pathologies or intestinal disorders”. In the manuscript is only "Enterocytozoon bieneusi is the mainly pathologies".

**Response:** Thank you very much. We have added all words “mainly pathologies or intestinal disorders”. Seen in Line 16.

**Summary and General Comments**

- Reviewer #1:

Dear Authors:

I want to thank the authors for the changes done and congratulate them for the work and research presented in the article. And I encourage them to continue in the same line for clearing doubts about the circulation and prevalence of this parasite.

**Response:** Thank you very much.

- Reviewer #3:

The author has already addressed the relevant questions and the authors have made the changes. I have just a few additional comments.

**Question 1:**1. “unusual dominant genotype NIA1” is missing in the abstract, please add it.

**Response:** Thank you very much. We have added this. Seen in Line 30 and 31.

**Question 2:**2. Line 59, ‘of which 96 have been…’ should be revised to ‘of which more than 96 have been…’

**Response:** Thank you very much. We have revised this. Seen in Line 59.

**Question 3:**3. Line 213, healthcare level also should be included here.

**Response:** Thank you very much. We have revised this. Seen in Line 213.

**Question 4:**4. Line 251, ‘…isolates identified in ruminants.’. Revised word ‘isolates’ to ‘genotypes’.

**Response:** Thank you very much. We have revised this. Seen in Line 251.
